# Psychological Sense of Community and the Use of Condoms and PrEP Among a Sample of Aging Black MSM

**DOI:** 10.1093/geroni/igaa057.3007

**Published:** 2020-12-16

**Authors:** Andre Brown, Mark Brennan-Ing, Steven Meanley, Sabina Haberlen, Deanna Ware, James Egan, Mackey Friedman, Michael Plankey

**Affiliations:** 1 University of Pittsburgh, Pittsburgh, Pennsylvania, United States; 2 Hunter College, CUNY, New York, New York, United States; 3 University of Pennsylvania, Philadelphia, Pennsylvania, United States; 4 Johns Hopkins University, Baltimore, Maryland, United States; 5 Georgetown University Medical Center, Washington, District of Columbia, United States

## Abstract

Psychological sense of community (PSOC) in Black men who have sex with men (BMSM) may facilitate condom and pre-exposure prophylaxis (PrEP) use to prevent HIV transmission. Understanding BMSM’s PSOC contribution to HIV risk reduction may inform HIV prevention efforts for this population, that is disproportionately affected by HIV. Adjusted for sociodemographic characteristics and HIV status, we conducted logistic regressions to test the association between PSOC and condom use among aging BMSM (n=176). Multivariate analyses exhibited no association between PSOC and condom use (AOR= 0.994, 95% CI= 0.942, 1.049). HIV+ participants had higher condom use odds compared to HIV- participants (AOR= 4.031, 95% CI= 1.723, 9.426). A sub-analysis of HIV- participants (n=61), showed no associated between PSOC and PrEP use (AOR= 1.002, 95% CI= 0.904, 1.112). These results have implications for secondary HIV prevention and future research on alternative aspects of social support that may increase BMSM’s HIV risk reduction behaviors.

